# Rubella seroprevalence among pregnant women in Burkina Faso

**DOI:** 10.1186/1471-2334-13-164

**Published:** 2013-04-04

**Authors:** Marc C Tahita, Judith M Hübschen, Zekiba Tarnagda, Da Ernest, Emilie Charpentier, Jacques R Kremer, Claude P Muller, Jean B Ouedraogo

**Affiliations:** 1 Institut de Recherche en Sciences de la Santé, 399 Avenue de la liberté, Bobo-Dioulasso, BP 545, Burkina Faso; 2 Institute of Immunology, Centre de Recherche Public de la Santé/Laboratoire National de Santé, 20A rue Auguste Lumière, Luxembourg, L, 1950, Luxembourg; 3 Clinique Lorentia, Ministère de la Santé, Bobo-Dioulasso, 01 BP 502, Burkina Faso

**Keywords:** Rubella, Seroprevalence, IgG, Burkina Faso

## Abstract

**Background:**

Despite the serious consequences of rubella infection during early pregnancy, very little is known about the rubella seroprevalence in a number of African countries including Burkina Faso.

**Methods:**

Between December 2007 and March 2008 serum samples were collected from 341 pregnant women in Bobo (n = 132, urban area) and Houndé (n = 209, rural area) and were tested for rubella-specific IgG antibodies with a commercial ELISA kit.

**Results:**

An overall seropositivity rate of 95.0% (324/341) was found, with a higher percentage in the urban population and in the oldest age group. Considering an antibody titer of at least 10 International Units per ml as protective, the overall immunity rate in the cohort of pregnant women was 93.3% (318/341).

**Conclusions:**

The high overall seropositivity rate in the absence of routine immunization suggests a continuous transmission of endemic rubella virus in Burkina Faso, posing a threat to non-immune pregnant women.

## Background

Rubella is normally a self-limiting febrile illness without significant long term morbidity [1]. Infection during pregnancy, however, may lead to miscarriage, fetal death or the birth of an infant with congenital rubella syndrome (CRS) [2]. It is estimated that worldwide more than 100 000 children with CRS are born each year [3]. Although the rubella seroprevalence among women in child-bearing age has been studied in several African countries [4-9], only one report from the Upper Volta region in Burkina Faso dating back to 1982 [10] and a recent study comprising only 100 women from a single location [11] are currently available to estimate the CRS risk in this country. Rubella vaccination is not included in the national immunization schedule in Burkina Faso and only few doses of vaccine are applied in the private sector. The aim of this study was to determine how many pregnant women are at risk of primary infection with rubella in a rural and urban area in the region of Bobo-Dioulasso, Burkina Faso.

## Methods

Venous blood samples were collected between December 2007 and March 2008 from 341 pregnant women in health centers in Bobo (n = 132, urban area) and Houndé (n = 209, rural area). The women’s age ranged from 16 to 42 years (mean: 25.7 ± 5.8 years), with more than half (58.9%) of them being 20–29 years old (Table 1). Informed consent to participate in the study was obtained from all women and a questionnaire comprising data on educational, marital, and pregnancy status, monthly income and history of previous exanthematous diseases was completed for each participant. All samples were transported to the laboratory on the day of collection for serum extraction and subsequent storage at -20°C.

**Table 1:** Rubella IgG ELISA results in relation to age and place of residence

	**Age groups (in years)**				**Place of residence**	
	**16-19**	**20-29**	**30-39**	**40-42**	**Bobo**	**Houndé**
**No. of positives**	51 (92.7%; 95% CI 85.7-99.7%)	194 (96.5%; 95% CI 94.0-99.1%)	73 (92.4%; 95% CI 86.5-98.3%)	6 (100%)	126 (95.5%; 95% CI 91.9-99.0%)	198 (94.7%; 95% CI 91.7-97.8%)
**No. of positives with ≥ 10 IU/ml**	49 (89.1%; 95% CI 80.7-97.4%)	192 (95.5%; 95% CI 92.6-98.4%)	71 (89.9%; 95% CI 83.2-96.6%)	6 (100%)	125 (94.7%; 95% CI 90.8-98.5%)	193 (92.3%; 95% CI 88.7-96.0%)
**No. of negatives**	4	7	5	0	6	10
**No. of equivocals**	0	0	1	0	0	1

The serum samples were screened manually for rubella-specific IgG antibodies using a commercial ELISA test (Enzygnost Anti-Rubella-Virus/IgG, Siemens, Germany). All equivocal samples were retested and if the result was confirmed, the sample was classified as equivocal, otherwise as positive or negative.

Statistical data analysis was done using the statistical package for social science software (SPSS, version 15, Chicago Incorporation), Data Analysis and Statistical Software (Stata, version 10, StataCorp LP, Texas, USA) and Microsoft Office Excel 2004. Tests of significance were conducted using the chi-square test at a significance level of 0.05. The study protocol was approved by the Institutional Ethics Committee of Centre Muraz.

## Results

All but 17 of the 341 pregnant women tested were rubella IgG positive, which corresponds to an overall seropositivity rate of 95.0% (95% CI 92.0-99.4%). Seropositivity was lowest among the 30–39 and the 16–19 year old women and highest among the 40–42 year olds (92.4%; 95% CI 86.5 – 98.3% and 92.7%; 95% CI 85.7-99.7% versus 100.0%) (Table 1). The seropositivity rate was higher among women from the urban than the rural community (95.5%; 95% CI 91.9-99.0% versus 94.7%; 95% CI 91.7-97.8%, p > 0.05). There were no statistically significant correlations between rates of rubella seropositivity and educational, marital, and pregnancy status, monthly income or history of previous exanthematous diseases.

Of the 324 seropositive women, 318 (193 or 97.5%; 95% CI 96.0-99.5% from the rural setting and 125 or 99.2%; 95% CI 98.2-99.8% from the urban area) had antibody titers of at least 10 International Units (IU) per ml and were thus considered protected against rubella infection [12], while the remaining 6 women are potentially at risk of reinfection. The overall immunity rate in the cohort of pregnant women was 93.3% (318/341, 95% CI 90.3-96.3%) with a higher percentage of immune women in the urban setting than in the rural area (94.7%; 95% CI 90.8-98.5% versus 92.3%; 95% CI 88.7-96.0%, p = 0.1) and in the oldest as compared to the youngest women (100% versus 89.1%; 95% CI 80.7-97.4%, p = 0.6) (Table 1).

## Discussion

This study provides for the first time rubella seroprevalence data for an urban and a rural setting in the western part of Burkina Faso. Though the study is limited to 341 pregnant women from two locations, the high overall immunity rate suggests a well-supported and continuous transmission of endemic rubella virus in the country. As a result most women become infected before reaching childbearing age. Nevertheless, the number of protected women continues to increase from 89.1% below the age of 20 to more than 95% in the 20–29 year olds, the age at which nearly 60% of the women gave birth. Beyond the age of 40 all women have antibodies against rubella. Since rubella vaccine is rarely given to adults in Burkina, our results suggest that a sizable number of women become infected during child-bearing age. In a previous report from Ouagadougou in central Burkina, an overall seroprevalence of 77% was found among pregnant women and the seropositivity rates seemed to decrease with age from 85% in the 18–25 year olds to 65% in the 36–50 year olds [11]. Nevertheless, the overall results indicate that a considerable proportion of pregnant women in Burkina are at risk of primary infection with rubella virus. In Burkina vaccination against rubella is provided by the private sector to a limited number of children. This low-coverage immunization may lead to a reduced circulation of rubella virus followed by a higher age of first exposure and therefore increased risk of CRS [13]. Based on the overall immunity rate found in this study and an estimated birth rate for Burkina Faso of 43.2 births/1000 population [14], we calculated that per year about 50000 pregnant women are at risk of rubella infection. Without any information on rubella incidence in Burkina Faso, reliable estimates of CRS case numbers are impossible, but considering that up to 90% of primary infections during the first 8–10 weeks of gestation may lead to fetal defects [12], the number of CRS cases is potentially quite high. Prevention of CRS is the main aim of rubella vaccination. Thus a rubella vaccination strategy in Burkina needs to ensure high enough vaccination coverage rates (≥ 80%) to interrupt indigenous virus circulation [12]. If this cannot be assured in a sustainable way the number of CRS cases may even further increase [13]. Similarly, vaccination in the private sector which does not reach high enough coverage can lead to more CRS cases [15].

## Conclusions

In conclusion, this study presents for the first time rubella seroprevalence data for an urban and a rural setting in the western part of Burkina Faso and although the study is restricted to the Bobo-Dioulasso region, the high seropositivity in the absence of routine vaccination suggests a continuous transmission of endemic rubella virus in Burkina Faso. While most women acquire seropositivity before reaching childbearing age, the virus represents a considerable threat to non-immune pregnant women.

## Abbreviations

CI: Confidence interval; CRS: Congenital rubella syndrome; ELISA: Enzyme-linked immunosorbent assay; IgG: Immunoglobulin G; IU: International units; ml: Milliliter; SPSS: Statistical package for the social sciences

## Competing interests

The authors declare that they have no competing interests.

## Authors’ contributions

MCT was involved in the design of the study, carried out most of the experimental work and drafted the article. JMH and ZT contributed to the conception of the study, the interpretation of the results and the writing of the manuscript. ED contributed to the recruitment of study participants and the management of their personal data. EC and JRK participated in the experimental work and the analysis and interpretation of the results. CPM and JBO have been involved in the initiation of the study and the revision of the manuscript. All authors read and approved the final manuscript.

## Pre-publication history

The pre-publication history for this paper can be accessed here:

http://www.biomedcentral.com/1471-2334/13/164/prepub
